# Supplementary material for: Direct on-swab metabolic profiling of vaginal microbiome host interactions during pregnancy and preterm birth
Source: Nat Commun. 2021 Oct 13;12:5967. doi: 10.1038/s41467-021-26215-w (PMC8514602; doi:10.1038/s41467-021-26215-w)
Supplement: Supplementary file 1 — Supplementary Information [file 41467_2021_26215_MOESM1_ESM.pdf]

Direct on-swab metabolic profiling of vaginal microbiome host interactions during pregnancy and preterm birth

Pamela Pruski,[1¥], Gonçalo D. S. Correia,[1,2¥], Holly V. Lewis[3,4,5], Katia Capuccini[3,4], Paolo Inglese[1,2], Denise Chan[3,4,5], Richard G. Brown[4,5], Lindsay Kindinger[6,], Yun S Lee[3,4], Ann Smith[7], Julian Marchesi[1,3], Julie A. K. McDonald[8], Simon Cameron,[1,9], Kate Alexander-Hardiman[1], Anna L. David[6], Sarah J. Stock[10], Jane E. Norman[10,11], Vasso Terzidou[3,4,12], T.G. Teoh[13], Lynne Sykes[3,4,5], Phillip R. Bennett[3,4,5,14], Zoltan Takats[1,2,3] and David A. MacIntyre[3,4,14*]*

SUPPLEMENTARY INFORMATION

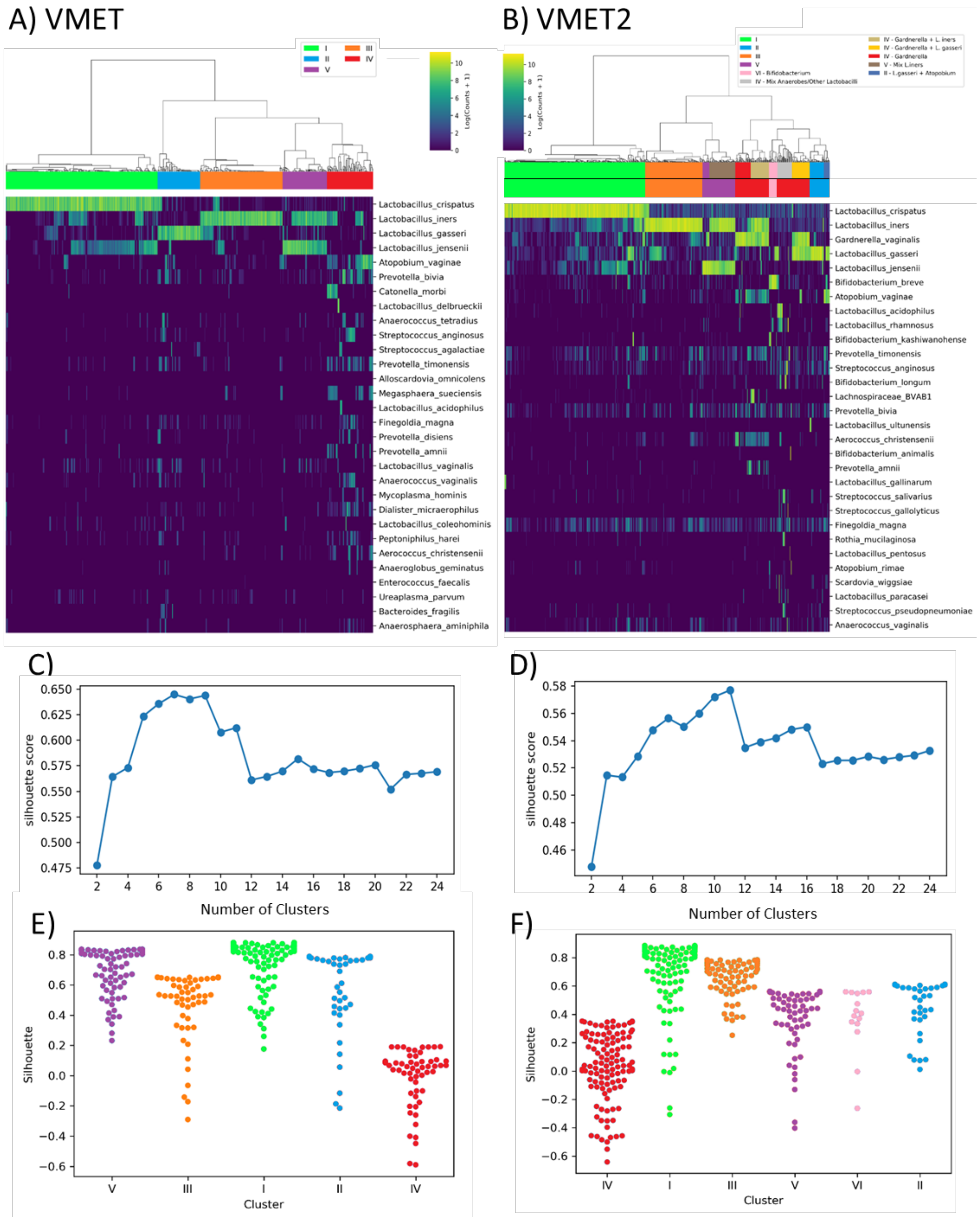

Supplementary Figure 1. Hierarchical clustering analysis of the 16*S* rRNA gene sequencing data from the VMET and VMET2 studies, and auxiliary diagnostic plots. A,B) Heatmap of log-transformed counts per microbial taxa found in the vaginal bacterial communities from the two patient cohorts. Hierarchical clustering of samples was based on their raw OTU counts aggregated (by summing) at species level, using Ward's method and the square-root of the Jensen-Shannon divergence as distance metric. C,D) Analysis of the average silhouette scores suggests an optimal (maximum average silhouette score) number of 11 and 9 clusters, for the VMET and VMET2 datasets, respectively. To facilitate comparison of results between the cohorts, clusters were coalesced into a smaller set of clusters (6 for VMET2 and 5 for VMET) consistent with previously described community state types: I = *L. crispatus* dominated; II = *L. gasseri* dominated; III = *L. iners* dominated; IV = mixed *Gardnerella vaginalis* + anaerobic bacteria; V = *L. jensenii* dominated; VI = *Bifidobacterium breve* dominated (VMET2 only); Sample specific silhouette score values representing how each sample fits into the final cluster in E) VMET and F) VMET2 (F). In both studies, silhouette scores were lowest for samples assigned to the CST IV cluster.

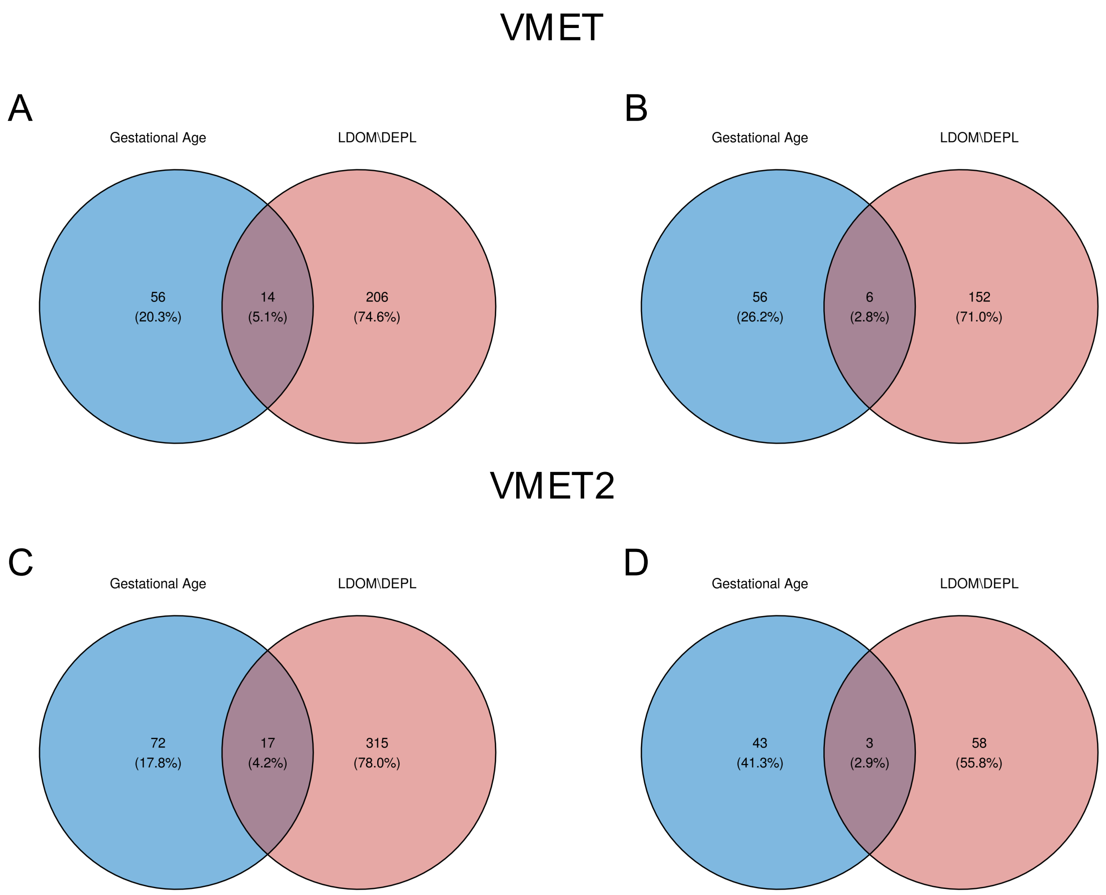

Supplementary Figure 2. Overlap of metabolic features between the microbiome (LDOM vs LDEPL) and gestational age signatures. Venn diagrams representing the numbers and proportion of features shared between the LDOM versus LDEPL and gestational age signatures, for different DESI-MS metabolic profiles in the VMET2 and VMET datasets. A) VMET DESI-MS negative mode signatures B) VMET DESI-MS positive mode C) VMET2 DESI-MS negative mode D) VMET2 DESI-MS positive mode. Numbers were calculated using the total number of significant features (after false discovery rate correction) found after the analysis of each cohort individually, before verifying the replication overlap across both cohorts.

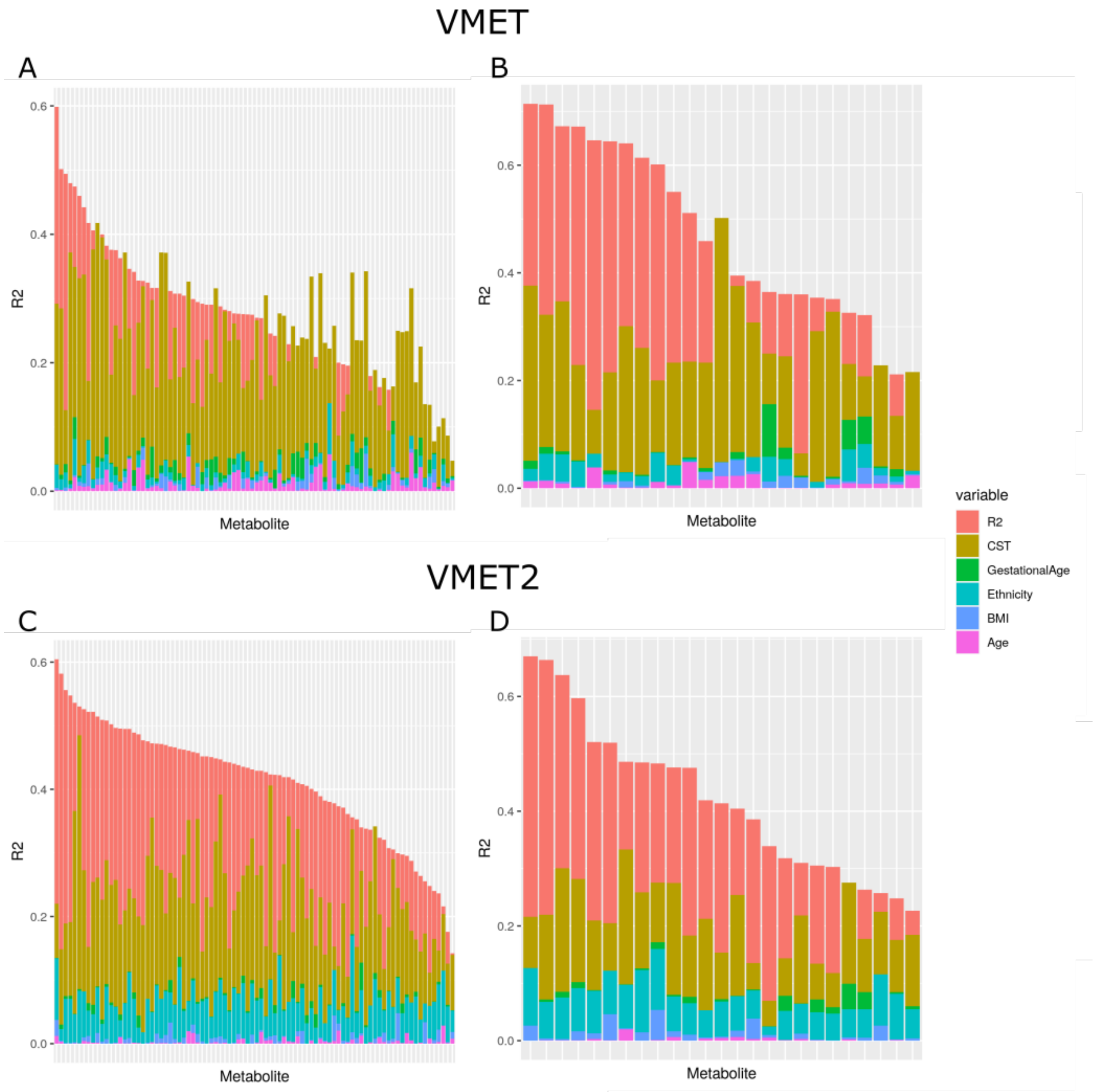

Supplementary Figure 3. Variance explained by main study factors in the microbial composition markers (LDOM vs LDEPL). Stacked-bar charts reporting the variance explained (r^2) by multiple factors for the DESI-MS features shown in Figure 2 B). R2 represents the conditional r^2 for mixed effect models (including proportion explained by individual), while CST, GestationalAge, Ethnicity, BMI, and Age report the semi-partial r^2 coefficients for each covariate. A) Features from VMET dataset, DESI-MS (-) metabolic profiles; B) VMET, DESI-MS (+) features; C) VMET2 dataset and DESI-MS (-) markers; D) VMET2, DESI-MS (+) features.

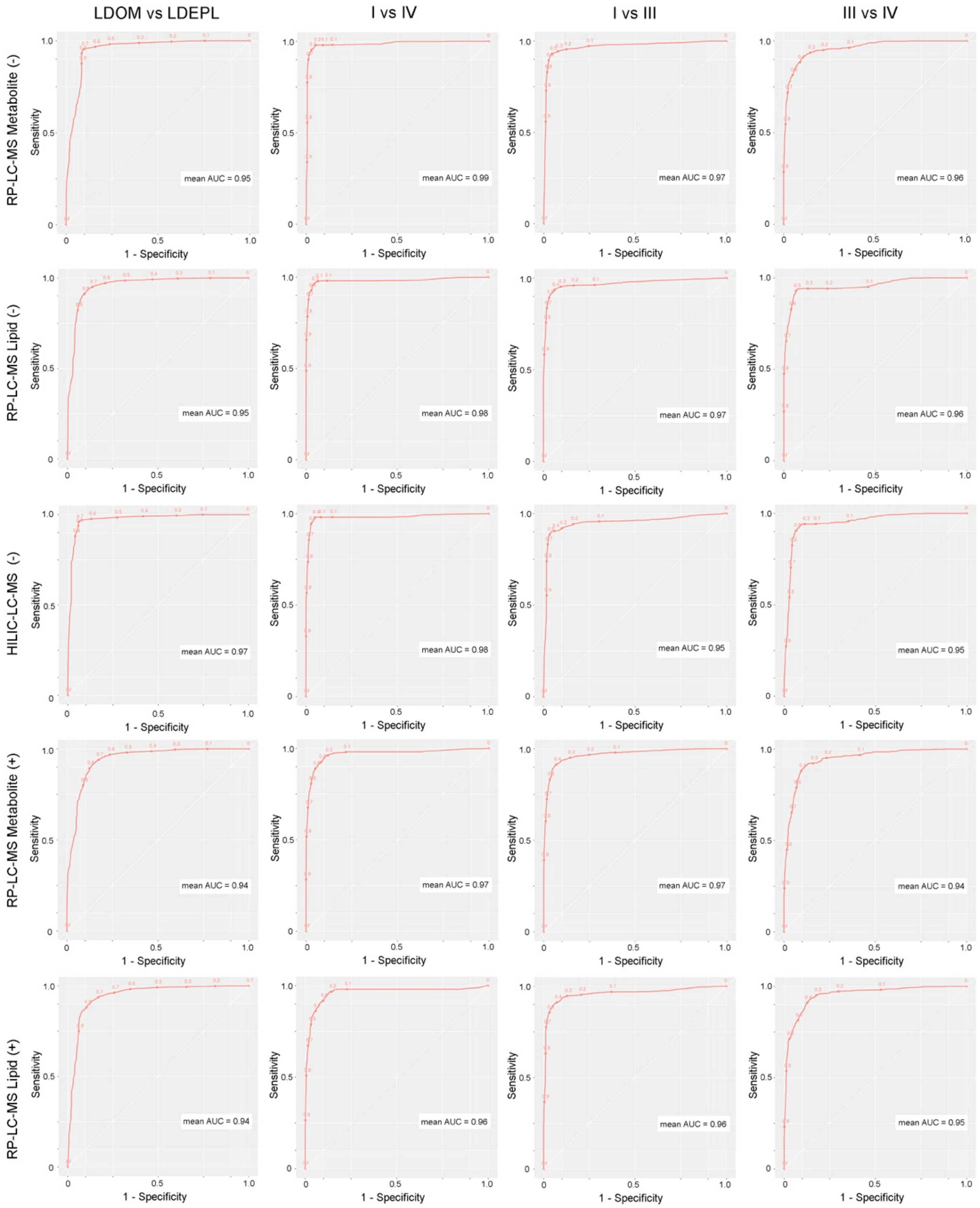

Supplementary Figure 4. ROC curve analysis plots and calculated AUC values for the random forest classifiers. Classifiers were trained to distinguish *Lactobacillus* spp.-dominated (LDOM) versus *Lactobacillus* spp.-depleted (LDEPL) (column 1) vaginal microbiome compositions using metabolic profiles obtained with different MS based assays (RP-LC-MS Metabolite (-) (row 1), RP-LC-MS Lipid (-) (row 2), HILIC LC-MS (-) (row 3), RP-LC-MS Metabolite (+) (row 4), RP-LC-MS Lipid (+) (row 5)). Similar analyses were performed between major CST groups; CST I versus CST IV (column 2), CST I versus CST III (column 3), and CST III versus CST IV (column 4).

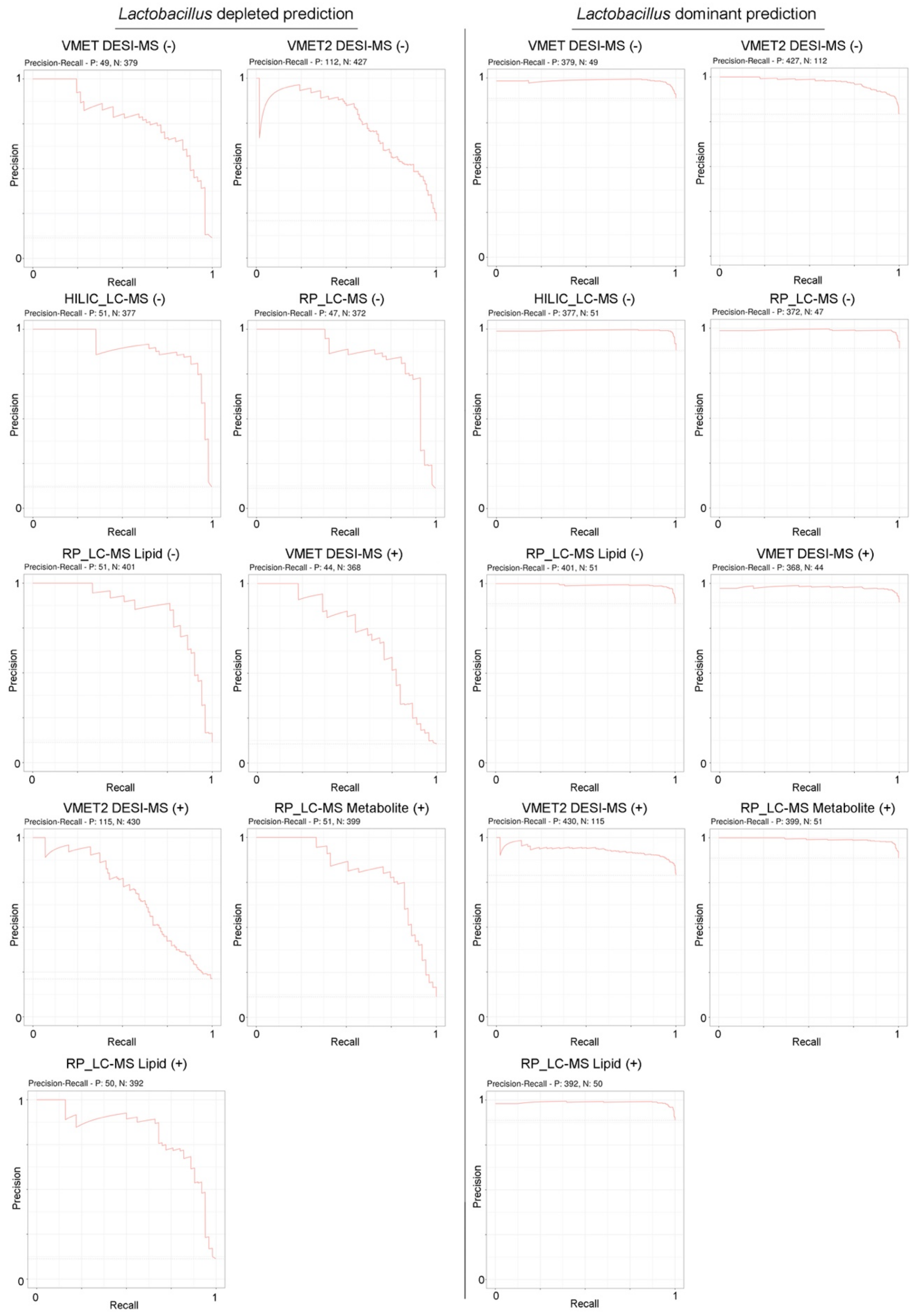

Supplementary Figure 5. Precision-Recall curve for *Lactobacillus* spp.-depleted (left) and *Lactobacillus* spp.-dominated(right) prediction obtained for all tested MS modes (DESI-MS +/-, HILIC-LC-MS -, RP-LC-MS Metabolite +/-, RP-LC-MS Lipid +/-). The precision-recall curves show good performance in the detection of *Lactobacillus* dominant status across the different types of metabolic profiling assays. While detection of the *Lactobacillus* depleted status has a poorer recall/sensitivity profile, it still retains a high specificity (> 0.75) within a wide range of cut-off values, a trend which is also observable across multiple assays.

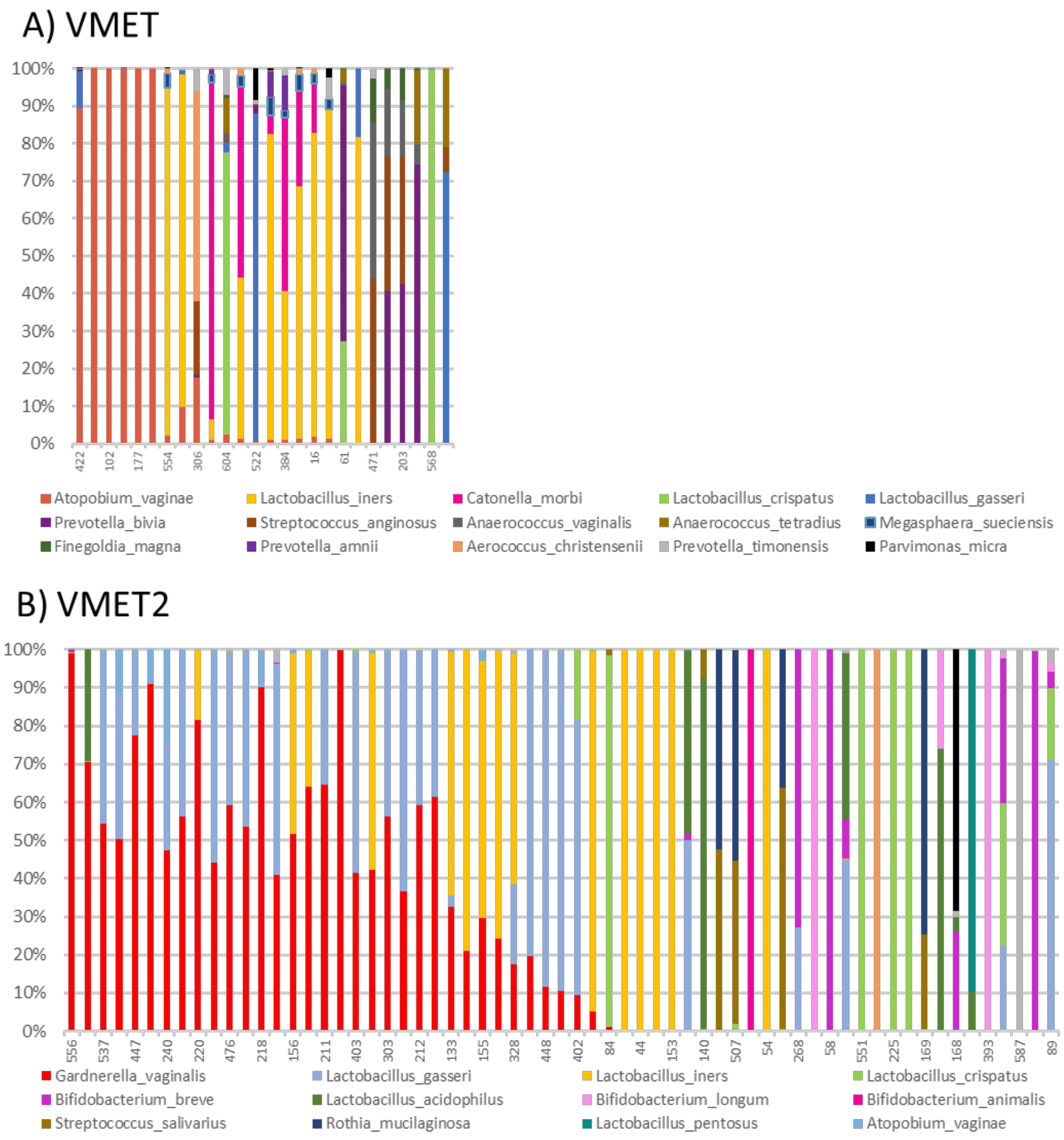

Supplementary Figure 6. Assessment of VMC in misclassified samples during LDOM versus LDEPL analysis by DESI-MS. A) Phylotype relative abundance of top 15 detected bacterial species with highest summed OTU counts observed in n = 26 misclassified samples by using 16S rRNA gene sequencing of vaginal mucosal swab samples in the VMET cohort. B**)** Phylotype relative abundance of top 15 detected bacterial species with highest summed OTU counts observed in n = 63 misclassified samples by using 16S rRNA sequencing of vaginal mucosal swab samples in the VMET2 cohort.

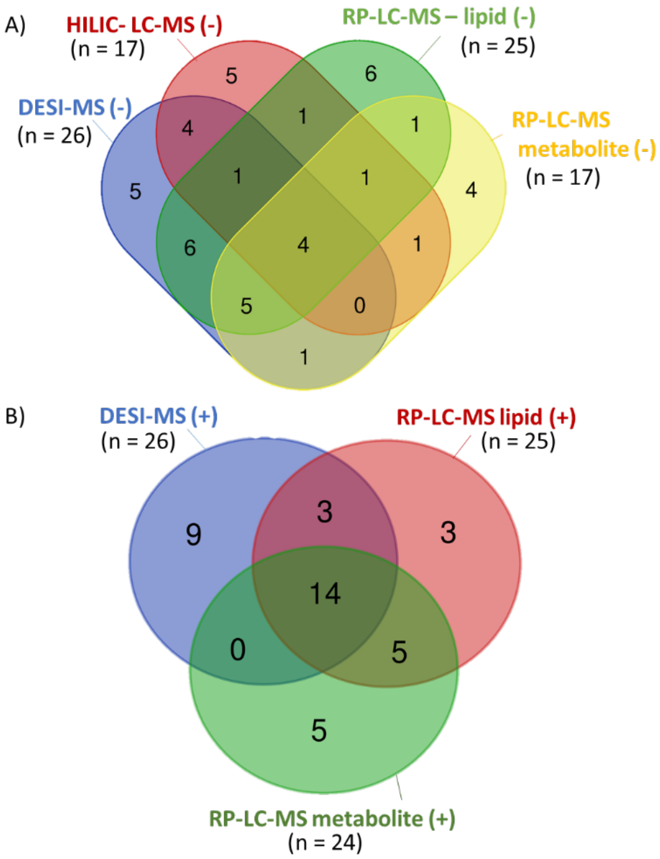

Supplementary Figure 7. Metabolite platform comparison of misclassified samples found during the LDOM versus LDEPL comparison in the VMET cohort. Venn diagram representing numbers of matching samples with same misclassified LDOM vs LDEPL status using different metabolic profiling assays (DESI-MS, HILIC-LC-MS, RP-LC-MS lipids, RP-LC-MS metabolites) using data obtained in both negative (A) and positive (B) ion mode.

Supplementary Table 1. Distribution of community state types (CST I, *L.crispatus*; CST II, *L. gasseri*; CST III, *L.iners*; CST IV, mixed anaerobes; CST V, *L. jensenii*; CST VI, *Bifidobacterium*; CST VII *Lactobacillus* other) according to ethnicity (Caucasian, Asian, Black, Others) and gestation at birth (<28, 28 – 34, 34 – 37, >37 weeks) in the VMET and VMET2 cohorts.

	CST species															
	Total population		CST I, *L.crispatus*		CST II, *L. gasseri*		CST III, *L. iners*		CST IV, mixed anaerobes		CST V, *L. jensenii*		CST VI, *Bifidobacterium*		CST VII, *Lactobacillus* other	
**VMET**																
n/N (%) subjects	160	(100%)														
n/N (%) swabs	455	(100%)	188	(41%)	53	(12%)	102	(22%)	51	(11%)	55	(12%)	NA	NA	6	(1%)
Ethnicity																
Caucasian	327/455	(72%)	133/327	(41%)	34/327	(10%)	75/327	(23%)	33/327	(10%)	47/327	(14%)	NA	NA	5/327	(2%)
Asian	33/455	(7%)	14/33	(42%)	6/33	(18%)	2/33	(6%)	4/33	(12%)	6/33	(18%)	NA	NA	1/33	(3%)
Black	93/455	(21%)	41/93	(44%)	11/93	(12%)	25/93	(27%)	14/93	(15%)	2/93	(2%)	NA	NA	0/93	(0%)
Gestation at birth																
< 28 weeks	9/455	(2%)	4/9	(44%)	0/9	(0%)	5/9	(56%)	0/9	(0%)	0/9	(0%)	NA	NA	0/9	(0%)
28 - 34 weeks	31/455	(7%)	12/31	(39%)	1/31	(3%)	12/31	(39%)	6/31	(19%)	0/31	(0%)	NA	NA	0/31	(0%)
34 - 37 weeks	39/455	(9%)	16/39	(41%)	7/39	(18%)	9/39	(23%)	0/39	(0%)	0/39	(0%)	NA	NA	0/39	(0%)
> 37 weeks	376/455	(83%)	156/376	(42%)	45/376	(12%)	76/376	(20%)	38/376	(10%)	55/376	(15%)	NA	NA	6/376	(2%)
**VMET2**																
n/N (%) subjects	205	(100%)														
n/N (%) swabs	573	(100%)	248/573	(43%)	34/573	(6%)	101/573	(18%)	107/573	(19%)	58/573	(10%)	15/573	(3%)	10/573	(2%)
Ethnicity																
Caucasian	373/573	(65%)	183/373	(49%)	20/373	(5%)	45/373	(12%)	60/373	(16%)	51/373	(14%)	6/373	(2%)	8/373	(2%)
Asian	71/573	(12%)	28/71	(39%)	7/71	(10%)	17/71	(24%)	10/71	(14%)	3/71	(4%)	6/71	(9%)	0/71	(0%)
Black	96/573	(17%)	26/96	(27%)	3/96	(3%)	34/96	(35%)	29/96	(30%)	1/96	(1%)	3/96	(3%)	0/96	(0%)
Others	33/573	(6%)	11/33	(33%)	4/33	(12%)	5/33	(15%)	8/33	(24%)	3/33	(9%)	0/33	(0%)	2/33	(6%)
Gestation at birth																
< 28 weeks	10/573	(2%)	2/10	(20%)	0/10	(0%)	1/10	(10%)	7/10	(70%)	0/10	(0%)	0/10	(0%)	0/10	(0%)
28 - 34 weeks	49/573	(9%)	24/49	(49%)	5/49	(10%)	11/49	(22%)	9/49	(18%)	0/49	(0%)	0/49	(0%)	0/49	(0%)
34 - 37 weeks	52/573	(9%)	29/52	(56%)	4/52	(8%)	5/52	(10%)	10/52	(19%)	1/52	(2%)	3/52	(6%)	0/52	(0%)
> 37 weeks	462/573	(80%)	193/462	(42%)	25/462	(5%)	84/462	(18%)	81/462	(18%)	57/462	(12%)	12/462	(3%)	10/462	(2%)

Supplementary Table 2. Summary of permutational multivariate analysis of variance (PERMANOVA) results. The proportion of metabolome variance explained (R^2) per covariate term (rows) is reported for all metabolic profiling datasets, alongside the degrees of freedom (Df), sum-of-squares (SumOfSqs), PERMANOVA Pseudo F-statistic (F), and P-value (Pr(>F)) for testing the null hypothesis that the covariate term has no effect on the metabolic profiles. Statistical testing was performed by sequential addition of the terms in the order displayed (CST to Subject ID). The "SubjectID" term captures the individual variability estimated from the repeated measures per subject, and "Residual" the remaining unexplained variance. Significance levels for the F-test P-value are highlighted as follows: "***" P-value < 0.001; "**" P-value < 0.01; "*" P-value < 0.05. 0.001 is the smallest numerical estimate possible for P-values using n=999 permutations.

	**VMET2 DESI-MS (-)**					**VMET DESI-MS (-)**				
	**Df**	**SumOfSqs**	**R^2**	**F**	**Pr(>F)**	**Df**	**SumOfSqs**	**R^2**	**F**	**Pr(>F)**
**CST**	6	178184.315	0.049	5.749	0.001***	5	84839.014	0.058	5.003	0.001***
**GestationalAge**	1	14874.142	0.004	2.879	0.001***	1	10466.357	0.007	3.086	0.001***
**Ethnicity**	7	86027.637	0.024	2.379	0.001***	2	8337.555	0.006	1.229	0.119
**BMI**	1	12270.672	0.003	2.375	0.003**	1	4249.339	0.003	1.253	0.145
**Age**	1	8373.041	0.002	1.621	0.024*	1	4635.687	0.003	1.367	0.097
**SubjectID**	193	1647443.099	0.452	1.652	0.001***	130	575187.857	0.396	1.305	0.001***
**Residual**	329	1699571.382	0.466			226	766433.795	0.527		
**Total**	538	3646744.289	1.000			366	1454149.604	1.000		
	**VMET2 DESI-MS (+)**					**VMET DESI-MS (+)**				
**CST**	6	99768.23	0.022	2.516	0.001***	5	77798.129	0.036	2.854	0.001***
**GestationalAge**	1	16155.44	0.004	2.444	0.001***	1	14590.057	0.007	2.676	0.002***
**Ethnicity**	7	94423.77	0.021	2.041	0.001***	2	16862.577	0.008	1.547	0.007**
**BMI**	1	14018.67	0.003	2.121	0.001***	1	6013.012	0.003	1.103	0.224
**Age**	1	13275.20	0.003	2.009	0.002**	1	6610.128	0.003	1.213	0.139
**SubjectID**	193	2031776.55	0.453	1.593	0.001***	128	835262.630	0.390	1.197	0.001***
**Residual**	335	2214087.29	0.494			217	1182976.812	0.553		
**Total**	544	4483505.14	1.000			355	2140113.344	1.000		
	**VMET LC-MS LPOS (+)**					**VMET LC-MS LNEG (-)**				
**CST**	5	288013.02	0.057	5.387	0.001***	5	514286.08	0.122	14.039	0.001***
**GestationalAge**	1	76991.97	0.015	7.200	0.001***	1	76966.43	0.018	10.505	0.001***
**Ethnicity**	2	46376.47	0.009	2.169	0.002**	2	37646.78	0.009	2.569	0.002**
**BMI**	1	28346.75	0.006	2.651	0.003**	1	15380.51	0.004	2.099	0.031*
**Age**	1	30734.90	0.006	2.874	0.001***	1	41333.00	0.010	5.641	0.001***
**SubjectID**	129	2002671.57	0.394	1.452	0.001***	130	1714376.15	0.405	1.800	0.001***
**Residual**	244	2609082.64	0.513			250	1831670.09	0.433		
**Total**	383	5082217.31	1.000			390	4231659.05	1.000		
	**VMET LC-MS RPOS (+)**					**VMET LC-MS RNEG (-)**				
**CST**	5	275468.380	0.224	28.918	0.001***	5	3186359.95	0.252	34.549	0.001***
**GestationalAge**	1	8264.662	0.007	4.338	0.004**	1	132518.04	0.010	7.184	0.001***
**Ethnicity**	2	9296.560	0.008	2.440	0.007**	2	83761.22	0.007	2.271	0.022*
**BMI**	1	4917.903	0.004	2.581	0.025*	1	57993.65	0.005	3.144	0.013*
**Age**	1	4220.610	0.003	2.215	0.041*	1	36044.08	0.003	1.954	0.080
**SubjectID**	130	455540.313	0.370	1.839	0.001***	129	5019428.00	0.397	2.109	0.001***
**Residual**	248	472487.431	0.384			223	4113319.54	0.326		
**Total**	388	1230195.859	1.000			362	12629424.49	1.000		
	**VMET LC-MS HNEG (-)**									
**CST**	5	2620517.42	0.187	20.937	0.001***					
**GestationalAge**	1	95588.73	0.007	3.819	0.004**					
**Ethnicity**	2	76361.74	0.005	1.525	0.092					
**BMI**	1	30249.67	0.002	1.208	0.227					
**Age**	1	54916.66	0.004	2.194	0.040*					
**SubjectID**	130	5413649.79	0.385	1.664	0.001***					
**Residual**	230	5757574.12	0.410							
**Total**	370	14048858.12	1.000							

Supplementary Table 3. Classification performance measures for assessing the VMC at genus and species levels using metabolomics profiling data obtained from the VMET and VMET2 cohort. A random forest classifier was used to discriminate between two classes for each contrast (LDOM vs LDEPL, I vs IV, I vs III and III vs IV), using as input the metabolomics data obtained from different MS platforms and modes (DESI-MS +/-, RP-LC-MS metabolites +/-, RP-LC-MS lipids +/-, HILIC-LC-MS -). A) Overview of sample and metabolite feature number used per mode for the classification performance analysis. B) Summary of predictive performance measures (Area under the curve (AUC), prAUC, accuracy, kappa, F1, sensitivity, specificity, positive predictive value (PPV), negative predictive value (NPV), prevalence, precision, recall, detection rate and balanced accuracy) for each tested contrast and mode. The values shown are the mean and one standard deviation, estimated from the repeated (15 repeats) 7-fold cross-validation test-sets.

A)		Sample size				Predictor feature number Used for all contrasts
Cohort	Mode	LD vs NLD	III vs IV	I vs III	I vs IV	
VMET	DESI-MS (-)	428	145	272	225	1333
VMET 2	DESI-MS (-)	539	198	332	338	1925
VMET	RP-LC-MS Metabolite (-)	419	142	267	219	12425
VMET	RP-LC-MS Lipid (-)	452	151	287	238	6269
VMET	HILIC-LC-MS (-)	428	146	270	226	13281
VMET	DESI-MS (+)	412	140	258	218	1555
VMET 2	DESI-MS (+)	545	199	334	335	1716
VMET	RP-LC-MS Metabolite (+)	450	153	287	236	4370
VMET	RP-LC-MS Lipid (+)	442	149	281	232	1299

B)	(VMET) DESI-MS (-)	(VMET2) DESI-MS (-)	RP-LC-MS Lipid (-)	RP-LC-MS Metabolite (-)	HILIC-LC-MS (-)	(VMET) DESI-MS (+)	(VMET2) DESI-MS (+)	RP-LC-MS Lipid (+)	RP-LC-MS Metabolite (+)
**LD vs NLD**									
AUC	94.1 ± 0.06	90.6 ± 0.04	96.1 ± 0.04	95.9 ± 0.05	97.7 ± 0.03	89.3 ± 0.09	83.1 ± 0.06	94.3 ± 0.05	94.8 ± 0.04
prAUC	79.3 ± 0.07	82.9 ± 0.05	80.4 ± 0.06	80.3 ± 0.07	82.9 ± 0.05	75.3 ± 0.09	77.6 ± 0.05	81.1 ± 0.06	77.8 ± 0.06
Accuracy	93.7 ± 0.02	87.7 ± 0.03	95.1 ± 0.03	95.7 ± 0.02	95.9 ± 0.02	93.1 ± 0.03	85.8 ± 0.03	94.2 ± 0.02	94.3 ± 0.02
Kappa	64.9 ± 0.14	57.2 ± 0.12	72.2 ± 0.16	78.0 ± 0.12	79.7 ± 0.1	55.5 ± 0.21	48.5 ± 0.13	64.5 ± 0.16	67.7 ± 0.14
F1	68.8 ± 0.13	64.2 ± 0.11	74.7 ± 0.14	80.4 ± 0.11	82.0 ± 0.09	59.4 ± 0.2	55.8 ± 0.12	67.2 ± 0.15	70.7 ± 0.13
Sensitivity	62.0 ± 0.16	54.5 ± 0.13	66.5 ± 0.17	79.6 ± 0.15	80.2 ± 0.12	49.2 ± 0.22	44.1 ± 0.13	57.5 ± 0.18	64.9 ± 0.17
Specificity	97.8 ± 0.02	96.4 ± 0.03	98.8 ± 0.02	97.7 ± 0.02	98.0 ± 0.02	98.4 ± 0.02	97.0 ± 0.02	98.9 ± 0.01	98.0 ± 0.02
PPV	80.7 ± 0.16	81.2 ± 0.12	89.2 ± 0.14	84.1 ± 0.13	86.0 ± 0.12	81.4 ± 0.22	80.3 ± 0.13	89.5 ± 0.13	81.4 ± 0.14
NPV	95.3 ± 0.02	89.1 ± 0.03	95.9 ± 0.02	97.5 ± 0.02	97.4 ± 0.02	94.2 ± 0.02	86.7 ± 0.03	94.9 ± 0.02	95.7 ± 0.02
Prevalence	88.8 / 11.2	78.7 / 21.3	88.8 / 11.2	88.8 / 11.2	88.8 / 11.2	88.8 / 11.2	88.8 / 11.2	88.8 / 11.2	88.8 / 11.2
Precision	80.7 ± 0.16	81.2 ± 0.12	89.2 ± 0.14	84.1 ± 0.13	86.0 ± 0.12	81.4 ± 0.22	80.3 ± 0.13	89.5 ± 0.13	81.4 ± 0.14
Recall	62.0 ± 0.16	54.5 ± 0.13	66.5 ± 0.17	79.6 ± 0.15	80.2 ± 0.12	49.2 ± 0.22	44.1 ± 0.13	57.5 ± 0.18	64.9 ± 0.17
Detection Rate	7.1 ± 0.02	11.3 ± 0.03	7.5 ± 0.02	8.9 ± 0.02	9.6 ± 0.01	5.2 ± 0.02	9.3 ± 0.03	6.5 ± 0.02	7.4 ± 0.02
Balanced Accuracy	79.9 ± 0.08	75.4 ± 0.07	82.7 ± 0.09	88.6 ± 0.07	89.1 ± 0.06	73.8 ± 0.11	70.5 ± 0.06	78.2 ± 0.09	81.4 ± 0.08
**I vs IV**									
AUC	96.4 ± 0.04	95.6 ± 0.03	98.5 ± 0.03	98.9 ± 0.02	98.4 ± 0.03	92.2 ± 0.06	87.7 ± 0.06	96.4 ± 0.05	97.1 ± 0.04
prAUC	85.2 ± 0.05	89.2 ± 0.03	82.7 ± 0.07	84.2 ± 0.07	85.5 ± 0.06	82.5 ± 0.06	82.6 ± 0.05	85.5 ± 0.05	85.8 ± 0.05
Accuracy	92.6 ± 0.04	87.8 ± 0.04	96.3 ± 0.03	96.6 ± 0.03	96.4 ± 0.03	91.4 ± 0.04	84.0 ± 0.04	93.0 ± 0.04	94.3 ± 0.04
Kappa	77.1 ± 0.15	70.5 ± 0.11	89.1 ± 0.1	90.4 ± 0.09	90.2 ± 0.08	73.9 ± 0.13	58.9 ± 0.12	79.1 ± 0.12	83.3 ± 0.12
F1	95.4 ± 0.03	91.3 ± 0.03	97.6 ± 0.02	97.8 ± 0.02	97.7 ± 0.02	94.6 ± 0.03	89.2 ± 0.03	95.6 ± 0.03	96.3 ± 0.03
Sensitivity	96.9 ± 0.04	92.4 ± 0.04	97.3 ± 0.03	96.9 ± 0.03	96.8 ± 0.03	96.6 ± 0.04	93.7 ± 0.04	96.1 ± 0.04	96.0 ± 0.04
Specificity	77.6 ± 0.17	77.2 ± 0.1	92.7 ± 0.09	95.6 ± 0.08	95.3 ± 0.07	74.0 ± 0.15	61.4 ± 0.13	81.9 ± 0.13	88.0 ± 0.12
PPV	94.1 ± 0.04	90.5 ± 0.04	98.1 ± 0.02	98.8 ± 0.02	98.7 ± 0.02	92.8 ± 0.04	85.3 ± 0.04	95.2 ± 0.03	96.7 ± 0.03
NPV	88.8 ± 0.12	82.1 ± 0.08	91.2 ± 0.1	90.5 ± 0.1	90.6 ± 0.09	87.7 ± 0.12	81.7 ± 0.11	86.7 ± 0.12	87.2 ± 0.12
Prevalence	41.3 / 11.2	41.3 / 18.7	41.3 / 11.2	41.3 / 11.2	41.3 / 11.2	41.3 / 11.2	41.3 / 11.2	41.3 / 11.2	41.3 / 11.2
Precision	94.1 ± 0.04	90.5 ± 0.04	91.2 ± 0.02	98.8 ± 0.02	90.6 ± 0.02	92.8 ± 0.04	85.3 ± 0.04	86.7 ± 0.03	96.7 ± 0.03
Recall	96.9 ± 0.04	92.4 ± 0.04	98.1 ± 0.03	96.9 ± 0.03	98.7 ± 0.03	96.6 ± 0.04	93.7 ± 0.04	95.2 ± 0.04	96.0 ± 0.04
Detection Rate	75.8 ± 0.03	64.5 ± 0.03	97.3 ± 0.03	76.1 ± 0.03	96.8 ± 0.03	74.4 ± 0.03	65.7 ± 0.03	96.1 ± 0.03	75.3 ± 0.03
Balanced Accuracy	87.2 ± 0.08	84.8 ± 0.06	76.4 ± 0.05	96.2 ± 0.04	74.9 ± 0.04	85.3 ± 0.08	77.6 ± 0.06	75.4 ± 0.06	92.0 ± 0.06
**I vs III**									
AUC	94.0 ± 0.05	97.2 ± 0.02	97.2 ± 0.04	97.3 ± 0.03	95.2 ± 0.03	80.0 ± 0.07	70.8 ± 0.07	97.2 ± 0.03	97.1 ± 0.03
prAUC	87.8 ± 0.05	91.2 ± 0.02	91.1 ± 0.04	90.8 ± 0.04	88.7 ± 0.03	74.1 ± 0.07	64.9 ± 0.06	91.1 ± 0.03	85.8 ± 0.03
Accuracy	89.4 ± 0.05	91.2 ± 0.04	94.3 ± 0.04	95.1 ± 0.04	93.7 ± 0.03	76.0 ± 0.06	72.9 ± 0.04	94.3 ± 0.04	94.3 ± 0.05
Kappa	76.6 ± 0.12	77.6 ± 0.1	87.3 ± 0.09	89.3 ± 0.08	86.0 ± 0.07	41.8 ± 0.14	21.0 ± 0.12	87.3 ± 0.09	83.3 ± 0.11
F1	91.9 ± 0.04	94.0 ± 0.03	95.7 ± 0.03	96.2 ± 0.03	95.1 ± 0.02	83.3 ± 0.04	82.9 ± 0.03	95.7 ± 0.03	96.3 ± 0.03
Sensitivity	92.8 ± 0.05	96.4 ± 0.03	96.5 ± 0.04	96.4 ± 0.04	96.0 ± 0.04	91.7 ± 0.05	93.5 ± 0.04	96.5 ± 0.05	96.0 ± 0.04
Specificity	83.2 ± 0.12	78.4 ± 0.11	90.2 ± 0.09	92.8 ± 0.08	89.4 ± 0.07	46.7 ± 0.12	23.9 ± 0.1	90.2 ± 0.09	88.0 ± 0.1
PPV	91.2 ± 0.05	91.8 ± 0.04	95.0 ± 0.04	96.2 ± 0.04	94.5 ± 0.03	76.5 ± 0.04	74.5 ± 0.03	95.0 ± 0.04	96.7 ± 0.05
NPV	86.8 ± 0.09	90.5 ± 0.08	93.7 ± 0.06	93.8 ± 0.07	92.9 ± 0.06	76.1 ± 0.15	62.6 ± 0.19	93.7 ± 0.07	87.2 ± 0.07
Prevalence	41.3 / 22.4	43.3 / 17.6	41.3 / 22.4	41.3 / 22 .4	41.3 / 22.4	41.3 / 22.4	41.3 / 22.4	41.3 / 22.4	41.3 / 22.4
Precision	91.2 ± 0.05	91.8 ± 0.04	93.7 ± 0.04	93.8 ± 0.04	92.9 ± 0.03	76.5 ± 0.04	74.5 ± 0.03	93.7 ± 0.04	87.2 ± 0.05

Recall	92.8 ± 0.05	96.4 ± 0.03	95.0 ± 0.04	96.2 ± 0.04	94.5 ± 0.04	91.7 ± 0.05	93.5 ± 0.04	95.0 ± 0.05	96.7 ± 0.04
Detection Rate	60.1 ± 0.03	68.5 ± 0.02	96.5 ± 0.03	96.4 ± 0.03	96.0 ± 0.02	59.7 ± 0.04	65.8 ± 0.03	96.5 ± 0.03	96.0 ± 0.03
Balanced Accuracy	88.0 ± 0.06	87.4 ± 0.06	62.9 ± 0.05	62.1 ± 0.04	62.2 ± 0.04	69.2 ± 0.07	58.7 ± 0.05	62.9 ± 0.05	75.3 ± 0.06
**III vs IV**									
AUC	92.9 ± 0.06	92.2 ± 0.05	96.2 ± 0.04	96.6 ± 0.03	96.1 ± 0.04	89.2 ± 0.08	86.4 ± 0.06	95.9 ± 0.05	95.0 ± 0.05
prAUC	81.9 ± 0.06	85.0 ± 0.06	85.7 ± 0.05	84.7 ± 0.04	84.7 ± 0.05	77.8 ± 0.08	79.2 ± 0.06	84.7 ± 0.05	83.8 ± 0.05
Accuracy	87.6 ± 0.06	85.8 ± 0.07	92.8 ± 0.06	90.8 ± 0.05	92.9 ± 0.06	83.7 ± 0.07	79.1 ± 0.07	88.5 ± 0.07	89.4 ± 0.06
Kappa	71.4 ± 0.15	71.5 ± 0.14	84.0 ± 0.12	78.9 ± 0.12	84.6 ± 0.12	63.2 ± 0.17	58.1 ± 0.13	74.2 ± 0.16	76.3 ± 0.14
F1	90.9 ± 0.04	85.1 ± 0.07	94.5 ± 0.04	93.1 ± 0.04	94.5 ± 0.04	87.7 ± 0.06	79.6 ± 0.06	91.3 ± 0.05	91.9 ± 0.05
Sensitivity	93.5 ± 0.07	85.2 ± 0.1	94.1 ± 0.06	93.6 ± 0.06	94.0 ± 0.06	90.8 ± 0.08	82.5 ± 0.09	91.1 ± 0.07	91.5 ± 0.07
Specificity	76.1 ± 0.16	86.3 ± 0.09	90.3 ± 0.11	85.0 ± 0.12	91.2 ± 0.11	70.9 ± 0.15	75.6 ± 0.11	83.3 ± 0.15	85.3 ± 0.13
PPV	88.9 ± 0.07	85.9 ± 0.08	95.3 ± 0.05	93.0 ± 0.05	95.4 ± 0.05	85.4 ± 0.07	77.5 ± 0.08	91.9 ± 0.07	92.9 ± 0.06
NPV	87.6 ± 0.12	86.8 ± 0.09	89.6 ± 0.1	88.0 ± 0.11	89.9 ± 0.1	82.8 ± 0.14	82.1 ± 0.08	83.8 ± 0.13	84.8 ± 0.11
Prevalence	22.4 / 11.2	17.6 / 18.7	22.4 / 11.2	22.4 / 11.2	22.4 / 11.2	22.4 / 11.2	22.4 / 11.2	22.4 / 11.2	22.4 / 11.2
Precision	88.9 ± 0.07	85.9 ± 0.08	89.6 ± 0.05	88.0 ± 0.05	95.4 ± 0.05	85.4 ± 0.07	77.5 ± 0.08	83.8 ± 0.07	84.8 ± 0.06
Recall	93.5 ± 0.07	85.2 ± 0.1	95.3 ± 0.06	93.0 ± 0.06	94.0 ± 0.06	90.8 ± 0.08	82.5 ± 0.09	91.9 ± 0.07	92.9 ± 0.07
Detection Rate	61.9 ± 0.05	41.3 ± 0.05	94.1 ± 0.04	93.6 ± 0.05	61.1 ± 0.04	58.4 ± 0.05	41.1 ± 0.04	91.1 ± 0.05	91.5 ± 0.05
Balanced Accuracy	84.8 ± 0.08	85.8 ± 0.07	62.3 ± 0.06	62.7 ± 0.06	92.6 ± 0.06	80.8 ± 0.09	79.1 ± 0.07	60.6 ± 0.09	61.0 ± 0.07

Supplementary Table 4. Statistically significant metabolite features found associated with host-related immune responses in cervicovaginal swab samples using DESI-MS data acquired in negative ion mode in the VMET2 patient cohort with reported ppm mass error, putative annotation, MS/MS and sub class of metabolic compounds. Measured *m/z* values highlighted in bold were additionally corrected by replacing value found in the sample with the highest measured *m/z* value in the patient cohort.

m/z measured	*m/z* theoretical	ppm error	Metabolite Annotation	Sum formula	Adduct	MS/MS	MSI level of annotation	Sub Class
192.0337	192.0336	-0.52	Lactoylcysteine	C6H11NO4S	M-H-	NO	3	Amino acids, peptides, and analogues
309.2799	309.2799	0.00	Phytenic acid (C20:1)	C20H38O2	M-H-	NO	3	Fatty acids and conjugates
321.2912	321.2911	-0.31	Heptadecyl-hydroxyimidazole	C20H38N2O	M-H-	NO	3	Imidazoles
337.3113	337.3112	-0.30	Docosenoic acid (C22:1)	C22H42O2	M-H-	NO	3	Fatty acids and conjugates
339.3268	339.3269	0.29	Docosanoic acid (C22:0)	C22H44O2	M-H-	NO	3	Fatty acids and conjugates
353.2805	353.2810	1.42	Octadecanoyloxamide	C20H38N2O3	M-H-	NO	3	NA
365.2808	365.2810	0.55	N-palmitoyl glutamine	C21H40N2O4	M-H2O-H-	NO	3	Amino acids, peptides, and analogues
366.2844	366.2844	-0.12	Isotope N-palmitoyl glutamine	(13C)C20H40N2O4	M-H2O-H-	NO	3	Amino acids, peptides, and analogues
375.2309	375.2308	-0.27	Hexadecenylsuccinic acid	C20H36O4	M+Cl-	NO	3	Dicarboxylic acids and derivatives
**376.2339**	376.2342	0.65	Isotope Hexadecenylsuccinic acid	(13C)C19H36O4	M+Cl-	NO	3	Dicarboxylic acids and derivatives
399.2684	399.2687	0.75	Oxosulfanyl-oxazolidinyl-octadecanamide	C21H40N2O3S	M-H-	YES	2	Carboximidic acids
400.2721	400.2721	-0.11	Isotope Oxosulfanyl-oxazolidinyl-octadecanamide	(13C)C20H40N2O3S	M-H-	NO	3	Carboximidic acids
413.2838	413.2843	1.30	Methyl-hexadecylcarbamothioylamino]-oxobutanoate	C22H42N2O3S	M-H-	NO	3	NA
427.2999	427.3000	0.23	Ethyl-hexadecylcarbamothioylamino-oxobutanoate	C23H44N2O3S	M-H-	NO	3	NA
455.3309	455.3313	0.88	Amino-sulfanylheptenyl-amino-oxooctadecanoic acid	C25H48N2O3S	M-H-	NO	3	NA
489.3148	489.3156	1.63	Dimethoxyphenyl-hexadecylcarbamothioyl-propenamide	C28H46N2O3S	M-H-	NO	3	NA
**682.5914**	682.5910	-0.46	Cer(d24:1/18:1)	C42H81NO3	M+Cl-	YES	2	Ceramides
**683.5948**	683.5944	-0.71	Isotope Cer(d24:1/18:1)	(13C)C41H81NO3	M+Cl-	NO	3	Ceramides
685.4988			Unknown			NO	3	
**885.5511**	885.5499	-1.31	PI(20:4/18:0)	C47H83O13P	M-H-	YES	2	Glycerophospholipid
**886.5540**	886.5533	-0.78	Isotope PI(20:4/18:0)	(13C)C46H83O13P	M-H-	NO	3	Glycerophospholipid
**896.5880**	896.5871	1.00	Galabiosylceramide (d18:1/16:0)	C46H87NO13	M+Cl-	YES	2	Ceramides
**897.5902**	897.5905	-0.30	Isotope Galabiosylceramide (d18:1/16:0)	(13C)C45H87NO13	M+Cl-	YES	2	Ceramides
**898.5856**	898.5850	0.63	Galbeta-Cer(d40:1)	C46H89NO11S	M+Cl-	YES	2	Ceramides

Supplementary Table 5. Classification performance measures for assessing the performance models using microbiome, metabolic, and immune profiling data for preterm birth prediction. A random forest classifier was used to discriminate between term and preterm birth at each sampling timepoint, using the centered-log-ratio transformed 16S data matrices, immune markers (VMET2 only) and DESI-MS +/- profiles. A) Overview of sample and feature number used for each model and comparison. B) Summary of predictive performance measures (Area under the curve (AUC), prAUC, accuracy, kappa, F1, sensitivity, specificity, positive predictive value (PPV), negative predictive value (NPV), prevalence, precision, recall, detection rate and balanced accuracy) for each tested assay and time point. The values shown are the mean and one standard deviation, estimated from the repeated (15 repeats) 7-fold cross-validation test-sets.

A)		Sample size			Predictor feature number
Cohort	Mode	1st Timepoint (weeks 0-14)	2nd Timepoint (weeks 14-24)	3rd Timepoint (weeks 24-40)	
VMET	16S data	47	141	112	80
VMET2	16S data	62	198	179	206
VMET2	Immune Markers	43	132	122	22
VMET	DESI-MS (-)	43	137	168	1333
VMET 2	DESI-MS (-)	59	192	168	1925
VMET	DESI-MS (+)	39	134	105	1555
VMET 2	DESI-MS (+)	57	193	174	1716

B)	VMET 16S data	VMET2 16S data	VMET2 Immune Markers	VMET DESI-MS (-)	VMET2 DESI-MS (-)	VMET DESI-MS (+)	VMET2 DESI-MS (+)
**PTB vs Term Birth – 1st Timepoint sample**							
AUC	32.37 ± 30.93	66.43 ± 24.35	68.87 ± 27.11	29.76 ± 30.12	59.76 ± 25.94	12.67 ± 19.4	50.86 ± 24.2
prAUC	38.39 ± 5.18	47.96 ± 8.74	46.82 ± 9.69	38.28 ± 5.24	47.86 ± 8.81	35.72 ± 3.37	44.16 ± 7.31
Accuracy	78.37 ± 8.82	80.57 ± 6.38	70.76 ± 12.11	80.68 ± 5.42	79.72 ± 4.67	87.81 ± 7.8	78.35 ± 5.92
Kappa	-0.66 ± 18.35	10.77 ± 26.43	2.69 ± 26.79	-1.14 ± 4.66	-0.14 ± 1.39	0 ± 0	-1.1 ± 4.6
F1	83.33 ± 19.25	70.18 ± 10.51	70.24 ± 13.36	NA	NA	NA	NA
Sensitivity	2.86 ± 15.24	10 ± 22.36	7.62 ± 20.55	0 ± 0	0 ± 0	0 ± 0	0 ± 0
Specificity	96.1 ± 8.64	99.18 ± 3.33	94.76 ± 10.84	98.86 ± 4.66	99.86 ± 1.39	100 ± 0	99.02 ± 4.17
PPV	17.39 ± 38.76	76 ± 43.59	38.57 ± 48.64	0 ± 0	0 ± NA	NA	0 ± 0
NPV	80.9 ± 6.08	80.82 ± 5.28	72.8 ± 8.81	81.44 ± 4.18	79.82 ± 4.69	82.93 ± 1.09	78.93 ± 5
Prevalence (PTB/Term)	19.15/80.85	20.97/79.03	27.91/72.09	18.6/81.40	20.34/79.66	12.82/87.18	21.05/78.95
Precision	17.39 ± 38.76	76 ± 43.59	38.57 ± 48.64	0 ± 0	0 ± NA	NA	0 ± 0
Recall	2.86 ± 15.24	10 ± 22.36	7.62 ± 20.55	0 ± 0	0 ± 0	0 ± 0	0 ± 0
Detection Rate	0.55 ± 2.79	2.04 ± 4.36	2.13 ± 5.5	0 ± 0	0 ± 0	0 ± 0	0 ± 0
Balanced Accuracy	49.48 ± 9.07	54.49 ± 11.48	51.19 ± 12.26	49.43 ± 2.33	49.93 ± 0.7	50 ± 0	49.51 ± 2.09
**PTB vs Term Birth – 2nd Timepoint sample**							
AUC	68.61 ± 17.18	53.06 ± 13.4	80.26 ± 10.6	57.37 ± 17.06	62.13 ± 10.86	52.24 ± 16.74	55.36 ± 13.31
prAUC	54.84 ± 9.04	50.3 ± 6.09	66.65 ± 6.18	50.53 ± 6.79	53.81 ± 5.37	48.42 ± 5.6	51.16 ± 6.05
Accuracy	81.4 ± 6.02	76.6 ± 5.26	76.23 ± 8.03	82.21 ± 4.44	79.7 ± 4.01	81.91 ± 3.76	78.45 ± 4.32
Kappa	10.34 ± 23.9	5.93 ± 17.01	31.61 ± 23.58	5.75 ± 19.21	4.52 ± 14	-1.29 ± 9.75	-1.09 ± 11.36
F1	44.38 ± 13.08	29.11 ± 9.29	48.81 ± 16.7	45.51 ± 9.3	30.22 ± 7.61	45.83 ± 8.33	27.72 ± 4.42
Sensitivity	13.97 ± 18.7	12.41 ± 12.5	36.63 ± 20.89	7.22 ± 14.67	6.92 ± 10.6	1.19 ± 6.05	3.46 ± 7.42
Specificity	94.53 ± 6.22	92.28 ± 5.9	91.7 ± 7.7	97.32 ± 3.8	96.5 ± 4.64	97.74 ± 3.98	95.62 ± 5.04
PPV	37.28 ± 41.18	30.32 ± 33.41	66.15 ± 29.12	35.12 ± 44.91	34.79 ± 41.08	10.61 ± 29.99	16.61 ± 31.99
NPV	85.02 ± 3.38	81.17 ± 2.52	79.08 ± 6.18	83.93 ± 3.02	81.81 ± 2.12	83.45 ± 1.8	81.2 ± 1.76
Prevalence (PTB/Term)	16.31/83.69	19.70/80.3	28.03/71.97	16.79/83.21	18.75 /81.25	16.42/83.58	18.65/81.35
Precision	37.28 ± 41.18	30.32 ± 33.41	66.15 ± 29.12	35.12 ± 44.91	34.79 ± 41.08	10.61 ± 29.99	16.61 ± 31.99
Recall	13.97 ± 18.79	12.41 ± 12.5	36.63 ± 20.89	7.22 ± 14.67	6.92 ± 10.6	1.19 ± 6.05	3.46 ± 7.42
Detection Rate	2.27 ± 3.08	2.48 ± 2.49	10.22 ± 5.84	1.2 ± 2.38	1.29 ± 1.96	0.2 ± 1	0.65 ± 1.4
Balanced Accuracy	54.25 ± 9.85	52.35 ± 6.92	64.17 ± 10.95	52.27 ± 7.63	51.71 ± 5.24	49.46 ± 3.71	49.54 ± 4.29
**PTB vs Term Birth – 3rd Timepoint sample**							
AUC	54.59 ± 20.27	56.77 ± 14.06	62.12 ± 13.05	46.38 ± 13.99	46.34 ± 14.82	65.67 ± 16.4	63.16 ± 14.64
prAUC	49.29 ± 7.58	51.07 ± 5.09	52.95 ± 6.51	47.74 ± 4.62	47.82 ± 5.1	52.69 ± 7.27	53.64 ± 7.02
Accuracy	77.37 ± 6.67	81.03 ± 4.02	73.96 ± 5.65	82.91 ± 2.9	82.44 ± 2.77	79.73 ± 4.99	82.87 ± 1.94
Kappa	2.48 ± 21.86	-4.27 ± 6.92	-4.44 ± 11.2	-1.59 ± 3.85	-2.49 ± 3.73	2.17 ± 16.27	-0.99 ± 6.08
F1	45.23 ± 11.99	30.16 ± 2.75	34.64 ± 4.85	NA	NA	45.13 ± 8.3	35.56 ± 3.85
Sensitivity	10.95 ± 20.52	0.71 ± 4.18	2.06 ± 7.29	0 ± 0	0 ± 0	6.19 ± 14.85	0.71 ± 4.18
Specificity	91.86 ± 7.71	95.93 ± 4.78	94.4 ± 6.76	98.77 ± 3.07	98.21 ± 2.76	95.84 ± 5.74	98.63 ± 2.27
PPV	21.82 ± 34.3	2.08 ± 9.02	8.78 ± 24.69	0 ± 0	0 ± 0	22.83 ± 33.03	6.67 ± 21.71
NPV	82.71 ± 4.13	83.87 ± 0.95	77.18 ± 2.75	83.76 ± 1.38	83.69 ± 1.41	82.4 ± 3.23	83.81 ± 0.69
Prevalence (PTB/Term)	17.86/82.14	15.64/84.36	22.95/77.87	16.07/83.93	16.07/83.93	18.10/81.91	16.09/83.91
Precision	21.82 ± 34.3	2.08 ± 9.02	8.78 ± 24.69	0 ± 0	0 ± 0	22.83 ± 33.03	6.67 ± 21.71
Recall	10.95 ± 20.52	0.71 ± 4.18	2.06 ± 7.29	0 ± 0	0 ± 0	6.19 ± 14.85	0.71 ± 4.18
Detection Rate	1.91 ± 3.5	0.11 ± 0.65	0.44 ± 1.54	0 ± 0	0 ± 0	1.19 ± 2.84	0.11 ± 0.67
Balanced Accuracy	51.41 ± 10.15	48.32 ± 2.96	48.23 ± 4.6	49.38 ± 1.53	± 1.38	51.01 ± 7.24	49.67 ± 2.24

Supplementary Table 6. **Bacterial isolates and culture conditions used for DESI-MS analysis.**

Isolate #	Genera	Species	Temp (ºC)	Atmosphere	Length (h)	Media	Specimen Type
DSM 15829	*Atopobium*	*Atopobium vaginae*	37	Anaerobic	48	CBA	Vagina
DSM 20213	*Bifidobacterium*	*Bifidobacterium breve*	37	Anaerobic	48	BA	Intestine of infant
DSM 4944	*Gardenella*	*Gardnerella vaginalis*	37	Anaerobic	48	CBA	Vagina
NHS 219	*Lactobacillus*	*Lactobacillus crispatus*	37	CO_2	48	CBA	Vagina
NHS 1712	*Lactobacillus*	*Lactobacillus crispatus*	37	CO_2	48	CBA	Vagina
NHS 1713	*Lactobacillus*	*Lactobacillus crispatus*	37	CO_2	48	CBA	Vagina
NHS 1757	*Lactobacillus*	*Lactobacillus crispatus*	37	CO_2	48	CBA	Vagina
NHS 1759	*Lactobacillus*	*Lactobacillus crispatus*	37	CO_2	48	CBA	Vagina
NHS 401	*Lactobacillus*	*Lactobacillus gasseri*	37	CO_2	48	CBA	Vagina
NHS 902	*Lactobacillus*	*Lactobacillus gasseri*	37	CO_2	48	CBA	Vagina
NHS 1272	*Lactobacillus*	*Lactobacillus gasseri*	37	CO_2	48	CBA	Vagina
NHS 1588	*Lactobacillus*	*Lactobacillus gasseri*	37	CO_2	48	CBA	Vagina
NHS 1758	*Lactobacillus*	*Lactobacillus gasseri*	37	CO_2	48	CBA	Vagina
DSM 13335	*Lactobacillus*	*Lactobacillus iners*	37	Anaerobic	48	MRS	Human urine
NHS 1339	*Lactobacillus*	*Lactobacillus jensenii*	37	CO_2	48	CBA	Endocervical
NHS 1342	*Lactobacillus*	*Lactobacillus jensenii*	37	CO_2	48	CBA	Vagina
NHS 1589	*Lactobacillus*	*Lactobacillus jensenii*	37	CO_2	48	CBA	Vagina
NHS 1693	*Lactobacillus*	*Lactobacillus jensenii*	37	CO_2	48	CBA	Vagina
NHS 2672	*Lactobacillus*	*Lactobacillus jensenii*	37	CO_2	48	CBA	Vagina
DSM 23384	*Prevotella*	*Prevotella amni*	37	Anaerobic	48	FAA	Amniotic fluid
NHS 987	*Prevotella*	*Prevotella disiens*	37	Anaerobic	48	CBA	Gastrointestinal tract
DSM 22865	*Prevotella*	*Prevotella timonensis*	37	Anaerobic	48	FAA	Human breast abscess
NHS 3836	*Staphylococcus*	*Staphylococcus aureus*	37	Aerobic	24	CBA	Blood
NHS 3929	*Streptococcus*	*Streptococcus agalactiae*	37	CO_2	48	CBA	Blood
NHS 4165	*Streptococcus*	*Streptococcus anginosus*	37	CO_2	48	CBA	Blood

CBA = Chocolate agar, BA = Blood agar, MRS = Man, Rogosa and Sharpe agar, FAA = Fastidious anaerobe agar
