# Supplementary material for: Direct on-swab metabolic profiling of vaginal microbiome host interactions during pregnancy and preterm birth
Source: Nat Commun. 2021 Oct 13;12:5967. doi: 10.1038/s41467-021-26215-w (PMC8514602; doi:10.1038/s41467-021-26215-w)
Supplement: Supplementary file 3 — Description of Additional Supplementary Files [file 41467_2021_26215_MOESM3_ESM.docx]

File name: Supplementary Data 1

Description: Annotation of metabolites identified by DESI-MS to discriminate between *Lactobacillus* spp.-dominated and *Lactobacillus* spp.-depleted vaginal microbiomes data. Metabolic features were identified using both negative and positive ion polarity modes in both VMET and VMET2 patient cohorts with ppm mass error, putative annotation, MS/MS and sub class of metabolic compounds reported. Statistical significance was assessed with linear mixed effect models (LME). The reported t-ratios and P-values were obtained from two-tailed Welch t-test comparisons between the grand mean of the CST I, II III, V and VII levels (LDOM group) and the mean of CST IV (LDEPL group). The Benjamini-Hochberg (BH) false discovery rate correction was applied to these P-values to select an LDOM vs LDEPL signature with an estimated FDR of 5% or less (q <0.05). Measured *m/z* values highlighted in bold were additionally corrected by replacing value found in the sample with the highest averaged measured *m/z* value in the patient cohort.
