# Supplementary material for: Direct on-swab metabolic profiling of vaginal microbiome host interactions during pregnancy and preterm birth
Source: Nat Commun. 2021 Oct 13;12:5967. doi: 10.1038/s41467-021-26215-w (PMC8514602; doi:10.1038/s41467-021-26215-w)
Supplement: Supplementary file 5 — Reporting Summary [file 41467_2021_26215_MOESM5_ESM.pdf]

nature research

Corresponding author(s): Zoltan Takats and David A. MacIntyre

Last updated by author(s): Sep 15, 2021

Reporting Summary

Nature Research wishes to improve the reproducibility of the work that we publish. This form provides structure for consistency and transparency in reporting. For further information on Nature Research policies, see our Editorial Policies and the Editorial Policy Checklist.

Statistics

For all statistical analyses, confirm that the following items are present in the figure legend, table legend, main text, or Methods section.

n/a	Confirmed	
☐	☒	The exact sample size (*n*) for each experimental group/condition, given as a discrete number and unit of measurement
☐	☒	A statement on whether measurements were taken from distinct samples or whether the same sample was measured repeatedly
☐	☒	The statistical test(s) used AND whether they are one- or two-sided<br>*Only common tests should be described solely by name; describe more complex techniques in the Methods section.*
☐	☒	A description of all covariates tested
☐	☒	A description of any assumptions or corrections, such as tests of normality and adjustment for multiple comparisons
☐	☒	A full description of the statistical parameters including central tendency (e.g. means) or other basic estimates (e.g. regression coefficient) AND variation (e.g. standard deviation) or associated estimates of uncertainty (e.g. confidence intervals)
☐	☒	For null hypothesis testing, the test statistic (e.g. *F*, *t*, *r*) with confidence intervals, effect sizes, degrees of freedom and *P* value noted<br>*Give P values as exact values whenever suitable.*
☒	☐	For Bayesian analysis, information on the choice of priors and Markov chain Monte Carlo settings
☐	☒	For hierarchical and complex designs, identification of the appropriate level for tests and full reporting of outcomes
☐	☒	Estimates of effect sizes (e.g. Cohen's *d*, Pearson's *r*), indicating how they were calculated

Our web collection on statistics for biologists contains articles on many of the points above.

Software and code

Policy information about availability of computer code

Data collection	The Waters MassLynx v4.2, Thermo XCalibur v 3.0, and ProteoWizard msconvert (v3.0.21105) software were used for mass spectrometry data collection.
Data analysis	The following software were used in the data analysis process:<br>R programming language v4.0.3 and the packages XCMS v3.10., maldiQUANT v1.19.3, lme4 (v1.1.25), emmeans (v1.5.2.1), pbkrtest (v0.4.8.6), r2glmm (v0.1.2), ggplot2 (v3.3.2), MuMIn (v1.43.17), vegan (v2.5.7), propr (v4.2.6), randomForest (v4.6-14), caret(v6.0-86) , pROC (v1.16.2), plotROC (v2.2.1), and precrec (v0.11.2);<br>Python programming language v3.8.5 and the nPYc-Toolbox v1.2.4, scipy v1.5.2, matptlotlib v3.3.2, and seaborn v0.11.0 libraries.

For manuscripts utilizing custom algorithms or software that are central to the research but not yet described in published literature, software must be made available to editors and reviewers. We strongly encourage code deposition in a community repository (e.g. GitHub). See the Nature Research guidelines for submitting code & software for further information.

Data

Policy information about availability of data

All manuscripts must include a data availability statement. This statement should provide the following information, where applicable:

- Accession codes, unique identifiers, or web links for publicly available datasets
- A list of figures that have associated raw data
- A description of any restrictions on data availability

The metabolic profiling data generated in this study have been deposited and made publicly available in the MetaboLights database under study identifier MTBLS717 [https://www.ebi.ac.uk/metabolights/MTBLS717]. The sequence data for the study are publicly available through the European Nucleotide Archive [https://www.ebi.ac.uk/ena] under accession numbers PRJEB 11895 (https://www.ebi.ac.uk/ena/browser/view/PRJEB11895), 12577 (https://www.ebi.ac.uk/ena/

browser/view/PRJEB12577) and (https://www.ebi.ac.uk/ena/browser/view/PRJEB41427). Relevant clinical and patient metadata is publicly available in the GitHub repository at https://www.github.com/gscorreia89/desims-cst-analysis/.

Field-specific reporting

Please select the one below that is the best fit for your research. If you are not sure, read the appropriate sections before making your selection.

- [x] Life sciences
- [ ] Behavioural & social sciences
- [ ] Ecological, evolutionary & environmental sciences

For a reference copy of the document with all sections, see nature.com/documents/nr-reporting-summary-flat.pdf

Life sciences study design

All studies must disclose on these points even when the disclosure is negative.

Sample size	No sample size calculations were performed. All available samples from both patient cohorts were used with replication of the main findings achieved in an independent patient cohort.
Data exclusions	DESI-MS swab spectra were excluded using 2 pre-established criteria based on spectrum quality: 1) high proportion (value higher than mean proportion + 2σ, estimated from all spectra) of total signal intensity originating from subset of background features (present in analyses of blank swabs, and therefore not part of the biological sample) and 2) low number of non-zero features (less than mean number of non-zero features - 2σ, estimated from all spectra).
Replication	For the main results in the paper relating to metabolome-microbiome associations, successful replication was performed using a second, independent patient cohort as described in the article. Replication of the immune-metabolome associations was not replicated due to unavailability of samples required.
Randomization	All samples used in the study were derived from two prospective, observational patient cohorts. There was no allocation to treatment or experimental groups. For metataxonomics and metabolomic profiling, samples were randomised prior to analysis to avoid correlations between run order and analytical batches and the main outcomes of interest (i.e. preterm and term gestation at delivery and CST type for metabolomics profiling).
Blinding	Sample collection was performed prospectively. Although no strict blinding protocol was used, metataxonomics and metabolomics profiling was performed without knowledge of outcomes.

Reporting for specific materials, systems and methods

We require information from authors about some types of materials, experimental systems and methods used in many studies. Here, indicate whether each material, system or method listed is relevant to your study. If you are not sure if a list item applies to your research, read the appropriate section before selecting a response.

Materials & experimental systems

n/a	Involved in the study	
x		Antibodies
x		Eukaryotic cell lines
x		Palaeontology and archaeology
x		Animals and other organisms
	x	Human research participants
x		Clinical data
x		Dual use research of concern

Methods

n/a	Involved in the study	
x		ChIP-seq
x		Flow cytometry
x		MRI-based neuroimaging

Human research participants

Policy information about studies involving human research participants

Population characteristics	Pregnant women over 18 years of age, with a singleton pregnancy, with and without risk factors for preterm birth. Women were considered at high risk of preterm birth either due to a history of previous sPTB, previous mid-trimester loss, recurrent miscarriage, incidental finding of cervical shortening and/or cervical excisional treatment.
Recruitment	Study participants were prospectively recruited at Imperial College Healthcare NHS Trust Hospitals (Queen Charlotte's and Chelsea and St Mary's Hospitals), London, UK, at Chelsea & Westminster Hospital (NHS Trust, London, UK), University College London Hospital (NHS Foundation Trust, London, UK) and the Royal Infirmary of Edinburgh, Scotland, UK. All study participants provided written informed consent prior to sampling and all experiments were performed in accordance with the approved institutional guidelines. Eligibility criteria were pregnant women with a singleton pregnancy, with and without risk factors for preterm birth. Exclusion criteria included women under 18 years of age, sexual intercourse within 72 h of sampling, vaginal bleeding in the preceding week, antibiotic use in the preceding 2 weeks, multiple pregnancies, HIV or Hepatitis C positive status. All eligible women were approached in order to prevent selection bias.
Ethics oversight	NHS National Research Ethics Service (NRES) Committees London - City and East (REC 12/LO/2003) and London–Stanmore (REC 14/LO/0328), and by the North of Scotland Research Ethics Service (REC 14/NS/1078).

Note that full information on the approval of the study protocol must also be provided in the manuscript.
